# Impact of Hydrogen-Enriched Solution Irrigation on Grain Yield and Nutritional Quality of Sweet Corn

**DOI:** 10.3390/foods15111847

**Published:** 2026-05-23

**Authors:** Hao Wang, Yuhao Wang, Ronghui Yu, Pengfei Cheng, Yan Zeng, Xu Cheng, Wenbiao Shen

**Affiliations:** 1 College of Life Sciences, Laboratory Center of Life Sciences, Nanjing Agricultural University, Nanjing 210095, China; 2 Air Liquide (China) R&D Co., Ltd., Shanghai 201108, China

**Keywords:** antioxidant capacity, grain yield, hydrogen-enriched water (HEW) irrigation, nutritional quality, sweet corn

## Abstract

Simultaneously improving the yield and, in particular, the nutritional quality of sweet corn (*Zea mays* L. *saccharata*), one of the most important cereal fresh foods worldwide, remains a major challenge. Here, we demonstrated that compared to control groups, hydrogen-enriched water (HEW) irrigation significantly improved agronomic performance, increasing kernel number (~10.55%) and ear length (~5.73%) while notably reducing barren tip length by about 60.73%. Regarding nutritional quality, HEW-treated kernels exhibited remarkable increases in soluble protein (~61.53%), total soluble sugars (~31.10%), vitamin C (~28.31%), total phenolics (~21.06%), and flavonoids (~40.56%). Micronutrients were also enhanced, such as zinc (~96.82%), iron (~51.70%), and manganese levels (~40.37%). HEW effectively modulated the expression of sugar metabolism-related genes. Specifically, the coordinated upregulation of key genes, such as *ZmSUS1* (~3.8 fold), *ZmINCW2* (~1.9 fold), and *ZmHXK1* (~1.6 fold), might contribute to the enhanced accumulation of sucrose (~11.79%), glucose (~6.21%), and fructose (~26.50%). Starch biosynthesis was also promoted. The improved sugar–acid ratio indicated enhanced taste quality. Importantly, representative key antioxidant genes (*ZmSOD2/4*, *ZmPOD1/2*, and *ZmCAT1/3*) as well as corresponding enzymatic activities in kernels were stimulated, which was negatively associated with lipid peroxidation. Overall, these results indicate that HEW irrigation is a promising, eco-friendly strategy that can be efficiently used to improve sweet corn yield and nutritional value.

## 1. Introduction

Sweet corn (*Zea mays* L. *saccharata*) is one of the most important cereal fresh foods worldwide, serving as a vital source of nutrients for human consumption and animal feed [1]. Distinguished by its high sugar content and favorable taste characteristics, sweet corn has gained increasing popularity in both fresh and processed markets [2]. However, modern sweet corn production faces serious challenges in simultaneously achieving high yield potential and superior nutritional quality, which are often competing objectives in agricultural society [3,4,5].

Current challenges in sweet corn production include several critical limitations. First, yield potential is frequently constrained by incomplete kernel development, manifesting as barren tip formation and reduced kernel numbers per ear, which cause negative impacts in commercial value and harvest efficiency. Second, nutritional quality parameters, including sugar content, protein level, and bioactive compound accumulation in particular, often require enhancement to meet consumer demands and nutritional requirements [6]. Third, micronutrient deficiencies in sweet corn, especially zinc and iron, represent a major concern for human nutrition, especially in regions where corn constitutes a dietary staple [7]. Finally, maintaining robust antioxidant capacity in kernels is essential, not only for post-harvest quality preservation but also for providing health-promoting compounds to consumers [8,9].

Traditional approaches to improving sweet corn yield and quality have relied primarily on genetic modification, optimized nutrient management, and various agronomic interventions. Conventional practices such as organic amendments have shown effectiveness in enhancing yield and quality [10]. However, these methods often face limitations including environmental concerns, inconsistent effectiveness across growing conditions, and potential soil health impacts. Moreover, achieving simultaneous improvements across multiple quality parameters remain challenging with conventional approaches alone. These parameters include yield, nutritional and mineral fortification, and antioxidant capacity.

Molecular hydrogen (H_2_) supplementation in agriculture may represent a novel and low-carbon biotechnological strategy applicable to the abundant production of crops, vegetables, and fruits in agri-food chains [11]. For example, ample evidence has demonstrated that hydrogen-enriched water (HEW) could influence growth and development, modulate metabolic pathways, and strengthen tolerance against environmental stimuli [12,13,14,15,16]. Notably, some studies have shown that hydrogen-based irrigation has potential to improve strawberry yield and shelf life by modulating rhizosphere microbial communities and enhancing nutrient uptake efficiency [15,17].

Importantly, growing evidence has reported hydrogen’s beneficial effects on food quality [11,18]. However, most previous studies for vegetables and fruits have focused on post-harvest stages [19], while systematic impacts of pre-harvest hydrogen treatments are relatively scarce, especially for sweet corn, one of the most important cereal fresh foods worldwide. In fact, sweet corn is a unique C4 cereal with a highly efficient photosynthetic carbon assimilation pathway, and, in particular, it relies on specialized mechanisms for endosperm sugar accumulation and sucrose-to-starch conversion [20]. For the important issues above, the present study adopted integrating morphological, biochemical, and molecular approaches to elucidate whether or how HEW irrigation influence substantial yield and nutritional value of sweet corn. Accordingly, our findings have theoretical and practical significance.

## 2. Material and Methods

### 2.1. Experimental Materials, Field Trials Location, Management, and Hydrogen Supply

The corn variety used in this experiment was “Shenxuetian 1” (*Zea mays* L. *saccharata*; bred by Tai’an Academy of Agricultural Sciences, Shandong Province, Tai’an, China).

In the growing season of 2024 (sowing on 11 September and harvesting on 18 November), the field trial experiments were conducted in the greenhouses (each was 6 m wide and 80 m long) at the Wanxiang Agriculture Base, Shanghai, China (longitude 121.88° east and latitude 30.98° north). This location for field trials provided a uniform and representative environment for sweet corn production, ensuring the extrapolation validity of the experimental results. The detailed meteorological data during the cultivation period, including daily temperature and precipitation, are comprehensively summarized in Supplementary Table S1. During the cultivation period, there was an average relative humidity of 65 ± 5%. While soil moisture was precisely managed via the drip irrigation system in the greenhouse, we acknowledge that external weather conditions, such as continuous precipitation, might indirectly affect sweet corn growth by reducing sunlight exposure.

A total of six independent greenhouses were utilized in this study, with three randomly assigned to the control (Con) group and three to the HEW treatment group to serve as true biological replicates (n = 3). To ensure representative sampling, each greenhouse was divided into two parts (each 6 m wide and 40 m long).

At the end of the growth period, sweet corn was harvested between 8:00 a.m. and 9:00 a.m. and promptly transferred to the laboratory. Within each independent greenhouse, a total of 30 ears were randomly collected using a five-point sampling method (15 ears per sub-zone). To balance the requirements of morphological evaluation and destructive biochemical assays, these harvested ears were allocated through a strict sub-sampling strategy:

(1) For phenotypic evaluation, 12 ears per greenhouse (6 ears from each sub-zone) were randomly evaluated for yield traits. The measurement values of these 12 ears were averaged to generate a single representative data point for that specific biological replicate. Among these, a subset of 3 representative ears per greenhouse was selected for phenotypic photography.
(2) For biochemical and molecular analyses, the remaining 18 ears per greenhouse were strictly allocated for destructive sampling. They were immediately threshed manually. Subsequently, the kernels from these 18 ears within the same greenhouse were randomly pooled and thoroughly mixed. A 100 g aliquot of this composite sample was then immediately flash-frozen in liquid nitrogen and stored at −80 °C to preserve biological characteristics and enzymatic activity.

To isolate the independent effect of hydrogen from confounding factors, such as nutrient and water conditions, an optimized local management practice was adopted. Fertilizers and pesticides were applied conventionally. An irrigation system was used throughout the growth period of sweet corn to maintain consistent and adequate soil moisture content based on real-time monitoring. To ensure the consistency of initial soil physicochemical properties, the experimental site was selected for its uniform soil texture and historical cropping uniformity. Prior to sowing, all six greenhouses underwent identical land preparation practices, including synchronized mechanized deep plowing and leveling to homogenize the topsoil layer. A uniform basal fertilization and irrigation regime were applied across all two parts to establish a standardized baseline environment, thereby minimizing potential confounding effects from soil spatial heterogeneity.

Hydrogen-enriched water (HEW) was prepared on-site by a hydrogen nanobubble water generator (Air Liquide (China) R&D Co., Ltd., Shanghai, China), and hydrogen gas was obtained from cylinders. To ensure a stable H_2_ concentration during the drip irrigation process (which lasted up to 5 h), HEW was continuously generated on-site. The dissolved hydrogen concentration was monitored in real-time both at the nanobubble water generator outlet and directly at the soil delivery point using a portable dissolved hydrogen meter (ENH-2000; TRUSTLEX, Osaka, Japan; calibrated by gas chromatography [17]) to ensure a stable concentration of molecular hydrogen (0.30–0.40 mM H_2_) in the HEW treatment group (HEW). The residence time of H_2_ in the above HEW was more than 12 h. The control group had an equal volume of surface irrigation water (Control group, Con). During the sowing and seedling stage, jointing stage, bell mouth stage, tasseling and silking stage, and filling stage, sweet corns were irrigated with HEW (drip irrigation, irrigation time: 0.5–5 h). In our drip irrigation system, the drippers were positioned closely adjacent to the soil surface. The physical dripping process of every droplet of HEW from the dripper to the soil took less than one second, ensuring that the volatilization or dissipation of dissolved hydrogen was negligible. This controlled hydrogen supply protocol was designed to simulate a standardized and reproducible hydrogen-based agricultural application scenario.

### 2.2. Measurement of Yield Traits

The average length, diameter, and barren tip length of 6 ears of corn per replicate were determined by ruler. From 5 ears of corn, all the kernels were removed to record the numbers of kernels for each ear. The fresh kernels (100 g) were then subjected to drying in a high-temperature oven (65 °C) until a constant weight was achieved. The dry weight was subsequently measured. The difference between the fresh and dry weights was used to calculate the water content. The measurement values of these sub-samples (per part) were then averaged to produce a single representative data point for specific biological replicate (n = 3).

### 2.3. Analysis of Protein, Sugar, and Starch Contents and Sugar–Acid Ratio

Soluble protein content was determined by using a BCA protein assay kit (Vazyme, Nanjing, China) according to the manufacturer’s instructions. Soluble sugars were extracted and measured using the anthrone–sulfuric acid method [21]. Absorbance was recorded at 620 nm, and glucose was used as the standard. Starch content was determined according to a previous method [22]. Contents of monosaccharide (fructose and glucose) and disaccharide (sucrose) were determined by HPLC (Agilent 1290, Agilent, Palo Alto, CA, USA), as described by Ressler [23]. Monosaccharide was analyzed directly, while polysaccharide was quantified after hydrolysis. Sugar–acid ratio was calculated by soluble sugar contents and organic acid contents [24].

### 2.4. Determination of Total Phenolic, Total Flavonoid, and Vitamin C Contents

Total phenolic content was determined using the Folin–Ciocalteu method [25]. Results were expressed as mg gallic acid equivalents per g fresh weight (mg·g^−1^ FW). Total flavonoids were quantified as previously described [26]. with rutin used for the standard curve. Results were expressed as mg·g^−1^ FW. Vitamin C content was determined using High-Performance Liquid Chromatography (HPLC, Infinity 1290, Agilent, Palo Alto, CA, USA [27]).

### 2.5. Analysis of Mineral Elements

The ion content of kernels was determined as follows [16]. Briefly, 0.2 g of dry sample was digested with nitric acid in a Microwave Digital Digestion System (Milestone Ethos T, Sorisole, Italy), and iron, manganese, zinc, and copper (Fe, Mn, Zn, and Cu) contents were measured with inductively coupled plasma mass spectroscopy (ICP-MS; NexION 2000, PerkinElmer, Waltham, MA, USA).

### 2.6. Antioxidant Capability and Lipid Peroxidation Assays

ABTS and DPPH radical scavenging activities were measured [25]. Data were expressed as percentage inhibitions. Ferric reducing antioxidant power (FRAP) was determined as described by Liu et al. [28]. Total reducing power of antioxidants (TRPA) was assessed using the potassium ferricyanide method [29]. Results were expressed as μmol FeSO_4_ equivalents per g FW.

The activities of SOD and CAT were determined as follows [30]. One unit (U) of SOD was considered to be the amount of enzyme required to inhibit 50% of nitroblue tetrazolium (NBT) reduction. For estimating CAT activity, the absorbance at 240 nm was recorded every 30 s for a total of 3 min, with an absorbance change of 0.01 per minute representing an enzyme activity unit (U). The activity of POD was determined as follows [31]. An absorbance change of 0.01 at 420 nm per minute was regarded as one unit (U). All enzyme activities were normalized to the soluble protein content and expressed as units per milligram of protein (U mg^−1^ protein).

Lipid peroxidation was evaluated by measuring thiobarbituric acid reactive substances (TBARS) according to Patterson et al. [32].

### 2.7. qPCR Analysis

Total RNA was extracted using a polysaccharide polyphenol plant total RNA extraction kit (DP441, Tiangen, Beijing, China). cDNA was synthesized by HiScript II Q RT SuperMix (R223, Vazyme, Nanjing, China). qPCR was performed on a Mastercycler ep^®^ realplex real-time PCR system (Eppendorf, Hamburg, Germany) with TransStart^®^ Top Green qPCR SuperMix (Transgen, Beijing, China). Primer sequences for qPCR were supplied in Supplementary Table S2. The 2^−ΔΔCT^ method [33] was used to quantify the relative-fold expression, which was analyzed using *ZmActin1* and *ZmTUB* [34] as the two reference genes.

### 2.8. Statistical Analysis

To ensure statistical rigor and avoid pseudo-replication, each scatter point in the bar charts represents the average data of samples obtained in two sub-samples within an independent greenhouse (i.e., three independent greenhouses per treatment, n = 3). All data are presented as the mean ± standard deviation (SD), and * indicates a significant difference between the two treatments (*t*-test, * *p* < 0.05, ** *p* < 0.01, *** *p* < 0.001, and ns means not significant).

The correlation analysis involving HEW, yield traits, sugar contents, nutritional metrics, micronutrient accumulation, antioxidant machinery, and related parameters was conducted using MetaboAnalyst 6.0 (available at https://www.metaboanalyst.ca; accessed on 7 January 2026). Prior to analysis, raw data underwent a series of preprocessing steps: normalization based on sample medians, cube-root transformation, and range scaling for data standardization.

## 3. Results

### 3.1. Yield Traits Were Improved by Hydrogen-Enriched Water Irrigation

**Figure 1:**
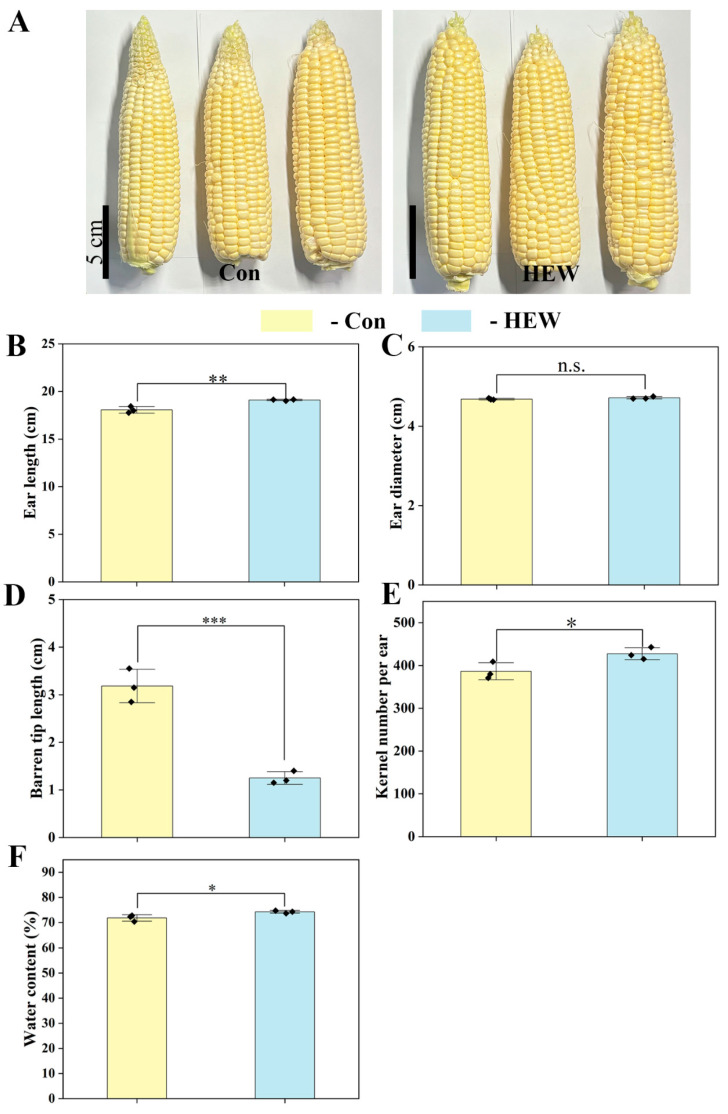
Morphological and yield traits of sweet corn. After harvesting, photography was taken (**A**), and ear length (**B**), ear diameter (**C**), barren tip length (**D**), kernel number per ear (**E**), and water content (**F**) in control (surface water irrigation, Con) and hydrogen-enriched water (HEW) irrigation groups were recorded (n = 3). All data are presented as the mean ± SD. *, **, and *** indicate significance at *p* < 0.05, *p* < 0.01, and *p* < 0.001; n.s., no significance.

Compared to the control group (Con; irrigation with surface water), the morphological characteristics of sweet corn after harvesting exhibited notable improvements achieved by hydrogen-enriched water (HEW) irrigation (Figure 1). For example, while there was no obvious alteration in ear diameter (Figure 1C), ear length was increased significantly, with 18.06 ± 0.48 cm in the control group compared to 19.10 ± 0.41 cm in HEW group, representing a 5.73% increase (*p* < 0.01; Figure 1B). Barren tip length, an important quality indicator, was decreased dramatically by 60.73%, with 3.18 ± 0.53 cm in the control compared to 1.25 ± 0.38 cm in the treated group (*p* < 0.001; Figure 1D), indicating better kernel development after HEW irrigation. Remarkably, the number of kernels per ear showed a substantial enhancement, rising from 386.6 ± 26.76 in the control group compared to 427.40 ± 14.94 under hydrogen treatment, corresponding to a 10.55% increase (*p* < 0.05; Figure 1E). It was further observed that compared to the control group, water content in the treatment group was increased by 3.36% (*p* < 0.05; Figure 1F), suggesting improved texture in the kernels by hydrogen. The above results clearly indicate that HEW irrigation could positively improve yield traits of sweet corn.

### 3.2. Accumulated Sugar and Starch in Sweet Corn Kernels

To address the knowledge gap regarding whether HEW simultaneously enhances multiple nutritional parameters, we systematically evaluated sugar profiles and starch contents, the primary determinants of sweet corn’s characteristic flavor and culinary properties [35].

**Figure 2:**
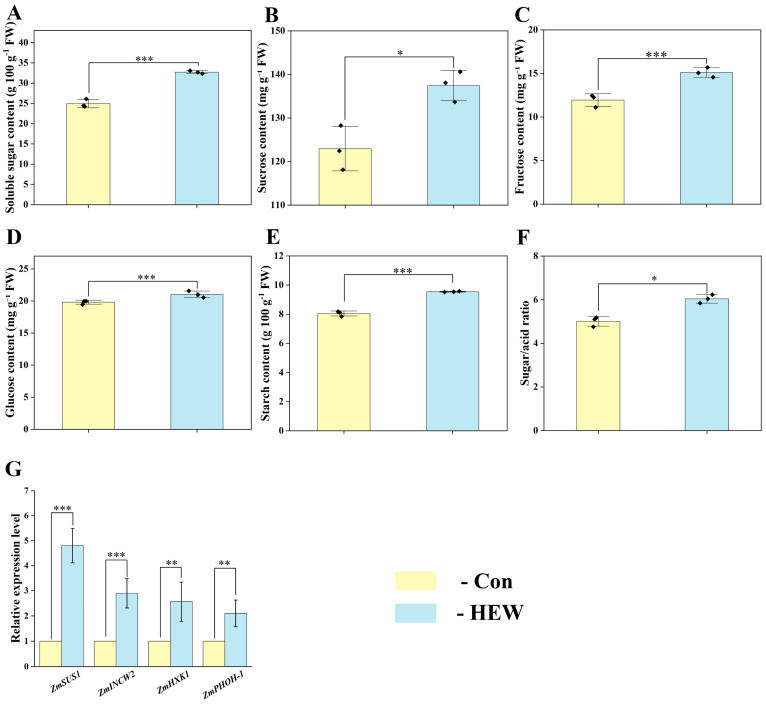
Improved carbohydrate accumulation after harvesting: total soluble sugar content (**A**); sucrose content (**B**); fructose content (**C**); glucose content (**D**); starch content (**E**); sugar–acid ratio (**F**); and relative expression levels of soluble sugar metabolism genes, including *ZmSUS1*, *ZmINCW2*, *ZmHXK1*, and *ZmPHOH-1* (**G**). All data are presented as the mean ± SD (n = 3; 0.2 g/treatment/repeat). *, **, and *** indicate significance at *p* < 0.05, *p* < 0.01, and *p* < 0.001.

Compared to those in the control group, sugar accumulation in HEW-irrigated sweet corn kernels was substantially enhanced, evaluated by the altered total soluble sugar, sucrose, fructose, glucose, and starch contents. For total soluble sugar, the most critical indicator of sweet corn quality, its content was elevated dramatically after HEW irrigation, compared to the control group (*p* < 0.001; Figure 2A). The content of sucrose, the predominant disaccharide responsible for sweet corn’s characteristic flavor profile, was also obviously increased (*p* < 0.05; Figure 2B). Similar significant changes were especially observed after subsequent evaluation of fructose, glucose, and starch contents (*p* < 0.001; Figure 2C–E). Notably, the sugar–acid ratio, a pivotal indicator of taste quality, was remarkably improved after HEW irrigation (*p* < 0.05; Figure 2F), further validating enhanced sweetness and flavor balance.

Further molecular evidence showed that representative genes functionally linked to soluble sugar metabolism were markedly stimulated by HEW irrigation, evaluated by the increased transcripts of *ZmSUS1*, *ZmINCW2*, *ZmHXK1*, and *ZmPHOH-1* (*p* < 0.01 or 0.001; Figure 2G). The above results matched with the alteration in carbohydrate composition achieved by HEW irrigation, especially in contents of sucrose, fructose, and glucose. Together, our results indicate molecular hydrogen control of carbohydrate accumulation simultaneously promotes metabolic gene expression and thus favors a suitable metabolic flux toward monosaccharide and disaccharide biosynthesis, both of which are beneficial for increasing sweet corn’s commercial value and consumer preference.

### 3.3. Nutritional Compounds Were Stimulated

**Figure 3:**
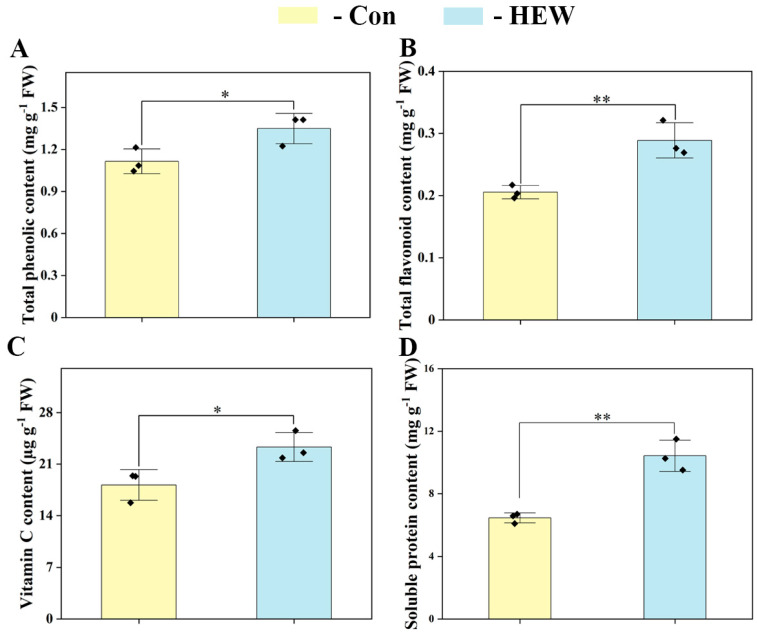
Nutritional compounds were increased. After harvesting, total phenolic (**A**), total flavonoid (**B**), vitamin C (**C**), and soluble protein (**D**) contents in kernels were determined. All data are presented as the mean ± SD (n = 3; 0.2 g/treatment/repeat). * and ** indicate significance at *p* < 0.05 and *p* < 0.01.

Nutritional compounds in sweet corn not only contribute to antioxidant capacity but also have potential health benefits for consumers. Among these, phenolics compounds, flavonoids, and vitamins represent key dimensions of functional food quality, extending beyond basic nutritional value to encompass health-promoting properties. Subsequent results showed that compared to the control group, HEW irrigation could significantly enhance phenolics compounds, flavonoids, vitamin C, and soluble protein in sweet corn kernels by 21.06% (*p* < 0.05; Figure 3A), 40.56% (*p* < 0.01; Figure 3B), 28.31% (*p* < 0.05; Figure 3C), and 61.53%, respectively (*p* < 0.01; Figure 3D).

### 3.4. Activation of Micronutrient Accumulation

**Figure 4:**
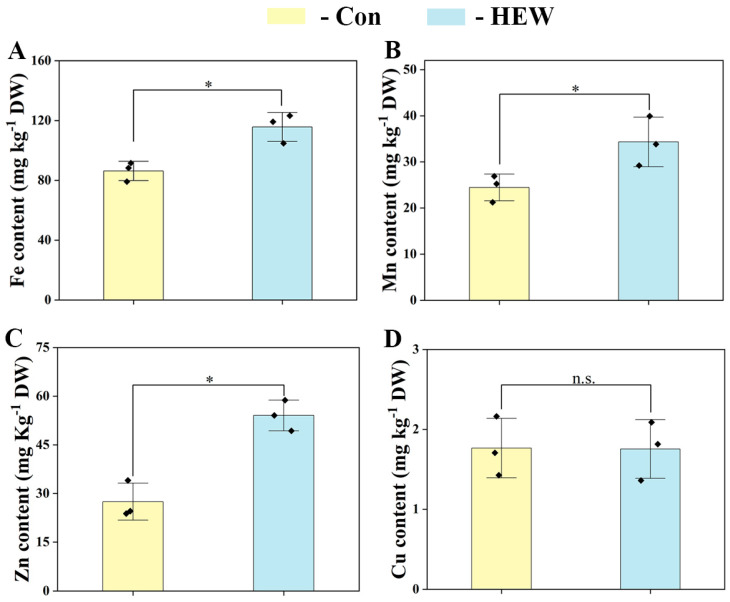
Contents of mineral elements were increased. After harvesting, iron (**A**), manganese (**B**), zinc (**C**), and copper (**D**) contents in kernels were determined. All data are presented as the mean ± SD (n = 3; 0.2 g/treatment/repeat). * indicate significance at *p* < 0.05; n.s., no significance.

Sweet corn, as a widely consumed fresh food, offers an ideal matrix for natural mineral fortification. To assess whether micronutrient accumulation in kernels is influenced by HEW, contents of iron, manganese, zinc, and copper were determined and compared. Interestingly, unlike the obviously unchanged copper level, HEW irrigation significantly enhanced iron, manganese, and zinc contents by 51.70%, 40.37%, and 96.82%, respectively (*p* < 0.05; Figure 4). These findings clearly indicate that hydrogen could stimulate micronutrient accumulation in sweet corn kernels.

### 3.5. Stimulated Antioxidant Machinery

Antioxidant systems in sweet corn might be functionally linked to maintain harvest duration and shelf life, thus contributing to nutritional value for consumers. To address this gap regarding the impact on comprehensive antioxidant systems, we evaluated both enzymatic defense mechanisms and non-enzymatic free radical scavenging capacity, both of which are directly or indirectly relevant to product quality and shelf-life stability.

**Figure 5:**
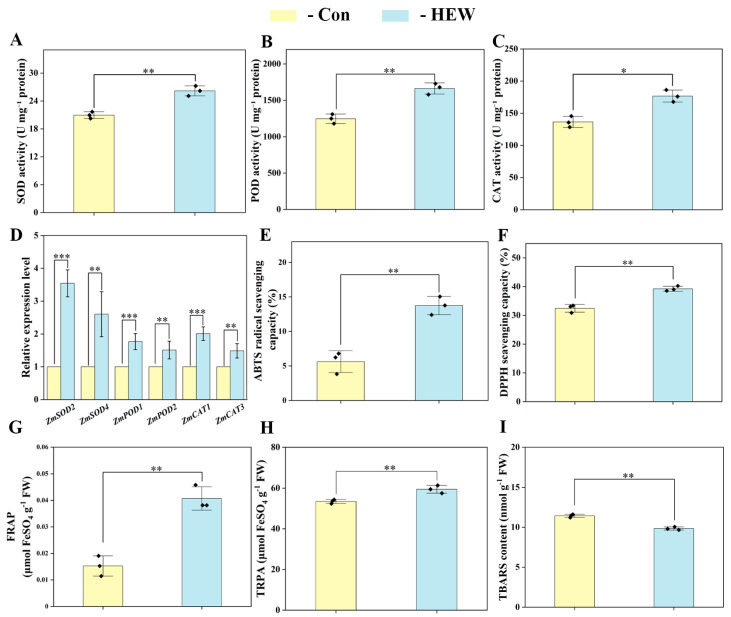
Antioxidant capability was driven by hydrogen-enriched solution irrigation. After harvesting, the representative antioxidant enzyme activities, including superoxide dismutase (SOD; (**A**)), peroxidase (POD; (**B**)), and catalase (CAT; (**C**)); the relative expression levels of antioxidant genes (*ZmSOD2*, *ZmSOD4*, *ZmPOD1*, *ZmPOD2*, *ZmCAT1*, and *ZmCAT3*; (**D**)); the non-enzymatic antioxidant capacities and lipid peroxidation, including ABTS radical scavenging capacity (**E**), DPPH scavenging capacity (**F**), ferric reducing antioxidant power (FRAP; (**G**)), and total reducing power of antioxidants (TRPA; (**H**)); and the levels of thiobarbituric acid reactive substances (TBARS; (**I**)) in kernels were analyzed. All data are presented as the mean ± SD (n = 3; 0.2 g/treatment/repeat). *, **, and *** indicate significance at *p* < 0.05, *p* < 0.01, and *p* < 0.001.

Compared to those in the control group, both enzymatic and non-enzymatic antioxidant capacities in HEW-irrigated sweet corn kernels were substantially enhanced, evaluated by the altered activities of key antioxidant enzymes and comprehensive antioxidant indicators. For the enzymatic antioxidant system, superoxide dismutase (SOD) activity was markedly elevated after HEW irrigation (*p* < 0.01; Figure 5A). The activities of peroxidase (POD) and catalase (CAT) also showed obvious enhancement (*p* < 0.01 or 0.05; Figure 5B,C). Corresponding genes, including *ZmSOD2*, *ZmSOD4*, *ZmPOD1*, *ZmPOD2*, *ZmCAT1*, and *ZmCAT3*, displayed similar increasing tendencies after HEW irrigation (Figure 5D). These results clearly reflect that molecular hydrogen regulated antioxidant enzymatic system at the enzymatic and transcriptional levels at least partially.

Regarding non-enzymatic antioxidant capacity, all measured indicators demonstrated significant improvements after HEW irrigation. For instance, ABTS radical scavenging capacity was dramatically increased (*p* < 0.01; Figure 5E). The DPPH scavenging capacity was also obviously enhanced (*p* < 0.01; Figure 5F). Similar changes were observed after subsequent evaluation of ferric reducing antioxidant power (FRAP) and total reducing power of antioxidants (TRPA) (*p* < 0.01; Figure 5G,H). Consistently, thiobarbituric acid reactive substances (TBARS) content, a pivotal indicator of lipid peroxidation, was remarkably reduced after HEW irrigation (*p* < 0.01; Figure 5I), further validating alleviated oxidative stress driven by molecular hydrogen.

Together, our results indicate that molecular hydrogen enhances antioxidant defense capacity by simultaneously promoting antioxidant gene expression and enzyme activities, thus favoring cellular redox homeostasis maintenance. Above alteration might be beneficial for improving sweet corn’s nutritional quality.

### 3.6. Correlation Analysis

**Figure 6:**
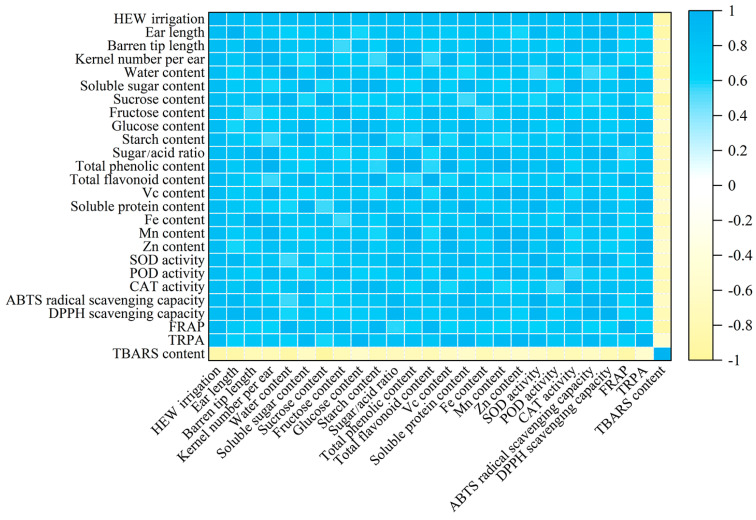
Correlation analysis among quality parameters, antioxidant capacities, and gene expressions, showing Pearson correlation coefficients among morphological traits, nutritional components, antioxidant indicators, and corresponding gene expression levels. The color scale represents the correlation coefficient (r), ranging from −1 (negative correlation, yellow) to +1 (positive correlation, blue). Only correlations with statistical significance are shown.

To explore the relevant relationships behind the quality improvement associated with HEW treatment, we conducted a Pearson correlation analysis between HEW application and most of the measured parameters. As shown in Figure 6, TBARS content was the parameter that displayed a strong negative correlation with HEW treatment; whereas most importantly, the majority of the other indicators (except ear diameter) showed strong positive correlations. This reflects the fact that HEW irrigation was significantly linked to these parameters. Overall, the above results suggest that HEW irrigation remarkably facilitates the possible relevant relationships of carbohydrate accumulation and antioxidant capacity, thereby contributing to an overall improvement in sweet corn quality. Certainly, the detailed relevant associations need to be further assessed by using a genetic approach.

## 4. Discussion

Sweet corn (*Zea mays* L.) is a globally significant vegetable crop, distinguished from other maize varieties by specific recessive alleles that result in reduced starch and increased sugar concentrations when harvested fresh [36]. Using sweet corn as the research material, our field trial experiments and nutritional evaluation found that both grain yield and nutritional quality of sweet corn were significantly enhanced after HEW irrigation. Our findings are significant for vegetable crop cultivation, and also particularly for related food production.

The first line of evidence supporting this conclusion is that morphological traits directly related to grain yield, and hereafter market quality, displayed remarkable enhancement after HEW irrigation, including increased ear length (Figure 1B, *p* < 0.01) and kernel number per ear (Figure 1E, *p* < 0.05), as well as decreased barren tip length (Figure 1D, *p* < 0.001). The pronounced reduction in barren tip length directly indicates improved kernel development and superior ear quality, two critical factors for commercial sweet corn production [37]. Similarly improved yield traits in lettuce, tomato, cucumber [38], rice [39], strawberry [15], blueberry [13], and cherry tomato [40] under normal growth conditions were previously reported in field observations. Morphological improvements could be achieved by molecular hydrogen in low-nitrogen conditions [12]. Therefore, these integrated results provide compelling evidence for hydrogen’s multifaceted benefits in producing premium-quality food or food materials, and also align with growing research supporting its role as a “green” solution [11].

Sweet corn’s market value and consumer acceptability are fundamentally determined by its composition of carbohydrates synthesized through its mutation in the starch synthesis pathway [41], particularly with respect to sweetness and starchiness [5,42]. Normally, sweetness is determined by the amount of total sugar in the endosperm, and sucrose is regarded as the most abundant and important sugar in sweet corn [41], while soluble sugar largely determines its quality [43]. Subsequent evaluation systematically illustrates the positive impacts of HEW irrigation on nutritional composition, mineral level, and antioxidant capacity. For example, our trail investigation showed that contents of carbohydrate compounds, including soluble sugar, sucrose, fructose, glucose, and starch in kernels, were significantly increased by HEW irrigation (Figure 2A–E). The above results were further functionally supported by the transcriptional regulation of sugar metabolism genes achieved by HEW, including *ZmSUS1* and *ZmINCW2* (Figure 2G), both of which individually encode sucrose synthase [44] and invertase [45]. We also noticed the up-regulation of both *ZmHXK1* (encoding hexokinase [46]) and *ZmPHOH-1* (encoding starch phosphorylase [47]) driven by hydrogen irrigation, reflecting the regulation complexity elicited by hydrogen. This needs further elucidation. Also, the sugar–acid ratio was positively influenced (Figure 2F). These findings clearly suggested the possibility that the sweetness of sweet corn in kernels were positively improved by molecular hydrogen, thus enhancing its quality [43]. In fact, unlike C3 crops investigated in the previous hydrogen-based agricultural studies, sweet corn utilizes a dedicated sucrose-to-starch conversion balance in the endosperm [48]. Our results demonstrate that HEW irrigation effectively modulates specific sugar metabolism genes (e.g., *ZmSUS1*, *ZmINCW2*) in this C4 system, highlighting a novel application of molecular hydrogen in regulating specialized carbon partitioning mechanisms.

Further results showed that both phenolic and flavonoid contents were increased significantly (Figure 3A,B), consistent with previous studies showing that molecular hydrogen, either supplied with HEW irrigation, hydrogen-rich water, or H_2_-modified atmosphere packaging, could significantly increase the total phenolic and flavonoid contents in grapes [49], strawberries [15], cherry tomatoes [40], fresh-cut kiwifruit [50] and fresh apple slices during storage [19]. As expected, soluble protein and vitamin C contents in kernels were positively influenced (Figure 3B,C). The above results clearly reflect the fact that the hydrogen has a potential role in increasing nutritional values, regardless of its application in pre-harvest and post-harvest stages of crop and horticultural products.

Ample evidence shows that micronutrients in foods play a central part in human metabolism and in the maintenance of tissue function; thus, they are beneficial for commercial and market values [51]. As shown in Figure 4, micronutrient accumulation in sweet corn kernels was substantially enhanced, particularly in iron, manganese, and zinc levels after HEW irrigation (Figure 4A–C), all of which are essential for human nutrition [52]. These findings were partially consistent with the previous results in field trials, illustrating that hydrogen-rich water irrigation increased iron and magnesium concentrations in rice seeds [39]. It is well documented that biofortification to enhance the micronutrient content and human health of staple crops through breeding programs, including conventional breeding or genetic manipulation, has largely focused on iron and zinc [53,54]. Accordingly, it is reasonably speculated that hydrogen-based irrigation, one of the innovative cultivation techniques, might be used in the fields of biofortification and sustainable agriculture in the near future.

Reestablishing redox homeostasis is a typical characteristic of the biological mechanism of hydrogen-based agriculture, thus enhancing the nutritional and market values of agricultural products, including shelf-life extension [13,18,55]. In hydrogen medicine, ample evidence similarly shows that H_2_ has global impacts on intracellular signaling molecules and their gene expression, all of which are mostly functionally linked to antioxidant, anti-inflammatory, and anti-apoptotic properties [56]. In our experimental conditions, it was clearly observed that after HEW irrigation, SOD, POD, and CAT activities and the corresponding gene expressions (*ZmSOD2*, *ZmSOD4*, *ZmPOD1*, *ZmPOD2*, *ZmCAT1*, and *ZmCAT3*) were significantly induced (Figure 5A–D). Other antioxidant indices, including ABTS radical scavenging capacity, DPPH scavenging capacity, FRAP, and TRPA, displayed the similar positive tendencies (Figure 5E–H). We also noticed that above antioxidant capabilities changes were strongly correlated with yield traits (except ear diameter) and nutritional quality improvement (Figure 6). Accordingly, decreased lipid peroxidation, assessed by changes in TBARS contents, was confirmed, when compared to the control group (Figure 5I). Importantly, this change showed a strong negative correlation with the majority of all indexes (Figure 6). This finding indicated that HEW irrigation apparently reduced oxidative damage in kernels, a primary cause of quality deterioration in fresh produce [19,57]. Importantly, above H_2_-driven antioxidant machinery, consistent with the activation of antioxidant enzyme and gene expression in wheat [14] and strawberry [58] after hydrogen supply during pre-harvest and post-harvest stages, is critical for maintaining quality from the field to the consumer, addressing significant benefit of hydrogen administration for commercial food supply chains [59].

Additionally, H_2_ has been shown to modulate soil microbial community structure [60,61], potentially contributing to improved nutrient availability and plant growth through enhanced soil microbial activity, as previously demonstrated in strawberry production where hydrogen-based irrigation significantly increased yield by altering rhizosphere microbial communities [15]. However, the lack of dynamic soil physicochemical and microbial data limits our understanding of these rhizosphere-mediated mechanisms. Future studies must integrate high-resolution soil analyses to fully characterize HEW-soil interactions and nutrient uptake.

We admit that the small sample size used herein, based on field trial experiments of a single cultivar during one growing season, relatively limits the wide application of the current conclusion. Nevertheless, our results successfully demonstrate that pre-harvest HEW irrigation could systematically alleviate the barrenness of sweet corn and improve its nutritional status, providing a promising ecological and environmental-friendly biotechnological strategy for the improvement of C4 crops.

It is worth mentioning that economic analysis confirms that the operational cost of hydrogen irrigation is remarkably low. According to the latest article, the price of green hydrogen is 35 RMB kg^−1^ [62]. At the target concentration of 0.4 mM H_2_ used in this study, the total expenditure for hydrogen gas over the entire growth cycle of sweet corn is approximately 2.47 USD per acre, even when considering a possible 90% escape loss and/or dissolution. When applied at a commercial scale, the capital cost of the hydrogen generator might be significantly decreased, especially in China [63]. As hydrogen production technologies continue to advance, HEW irrigation stands as a sustainable, scalable, and economically viable strategy for enhancing C4 crop yield and quality in modern agriculture.

## 5. Conclusions

**Figure 7:**
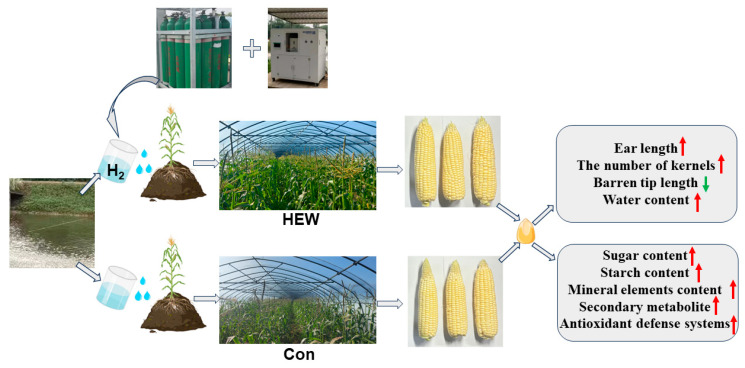
Schematic model for improving the yield and nutritional quality of sweet corn using HEW irrigation. The up and down direction of the red and green arrows represent the promotion or reduction effects achieved by HEW compared to the control (Con). HEW: hydrogen-enriched water; Con: control group (surface water).

Taken together, the positive effects of HEW (generated by hydrogen cylinder) on yield traits and nutritional quality of sweet corn were investigated. Further results showed that HEW irrigation not only increased ear length and kernel number while decreasing barren tip length, but it also positively improved the contents of soluble sugar, sucrose, fructose, glucose, starch, mineral elements (iron, manganese, and zinc), and nutritional compounds (total phenolics, flavonoids, vitamin C, and proteins) in kernels. The activation of antioxidant machinery was also observed (Figure 7). Importantly, the stimulation in carbohydrate accumulation and antioxidant capability via transcriptional activation of sugar metabolism genes and antioxidant genes was functionally involved. These findings might provide an important approach in sweet corn production for the quality improvement achieved by H_2_ application. Future sweet corn field trails may seek the application of a low-carbon photovoltaic hydrogen, with the aim of further exploring its potential practical value of green hydrogen in agricultural production.

## Data Availability

The original contributions presented in this study are included in the article/Supplementary Materials. Further inquiries can be directed to the corresponding author.

## Supplementary Materials

The following supporting information can be downloaded at: https://www.mdpi.com/article/10.3390/foods15111847/s1, Table S1. Summary of weather and environment from 11 September to 18 November. Table S2. Primer sequences of genes for qPCR detection.
