# Supplementary material for: Impact of Hydrogen-Enriched Solution Irrigation on Grain Yield and Nutritional Quality of Sweet Corn
Source: Foods. 2026 May 23;15(11):1847. doi: 10.3390/foods15111847 (PMC13256415; doi:10.3390/foods15111847)
Supplement: Supplementary file 1 [file foods-15-01847-s001.zip › Supplemental Table S2.pdf]

Supporting Material

Supplemental Table S2. Primer sequences of genes for qPCR detection

Gene name	Forward primer (5′ to 3′) and Reverse primer (5′ to 3′)	Accession number
ZmActin1	CATGGAGAACTGGCATCACACCTT(F)<br>CTGCGTCATTTTCTCTCTGTTGGC(R)	NM_001155179.2
ZmTUB	CACTGATGTTGCTGTCCTGC(F)<br>CGCTGTTGGTGATTTCGG(R)	NM_001174192.2
ZmCAT1	TGGATCCATACAAGCACCGC(F)<br>CATGCACAACACGTTCAGGG(R)	NM_001254879.2
ZmCAT3	GGATCCTACCAAGTTCCGTCC(F)<br>CTCGTTGTCGTTCCACACAG(R)	NM_001363892.1
ZmPOD1	GACTGCTTCGTCAGGGGTTG(F)<br>CCTCCAGTACGTCGAAGGC(R)	NM_001159274.2
ZmPOD2	TTTCCATTGTTGCGGACAGC(F)<br>GAAGCAGTCGTGGAAGTGGA(R)	NM_001254790.2
ZmSOD2	TGCATTCAGCTACGATCACA(F)<br>TGGTGCCCTTGACATCAGTG(R)	NM_001111865.2
ZmSOD4	CCGTGTTGCTTGTGGGATCA(F)<br>GCTCATCGGGTGCTTAATGG(R)	NM_001112234.2
ZmPHOH-1	TGCAGCGCATCACTTCAGA(F)	NM_001154922.2

	TGATCTCCTTCTCGGTGGTCT(R)	
ZmSUS1	TGGAAGGGTTGGATGCTATTG(F)<br>CCACCTTGGCATCTTTGTTC(R)	NM_001111853.1
ZmINCW2	GGCTACATCGGCAACTACGA(F)<br>TCCACGAACACGAACACCTC(R)	NM_001305860.1
ZmHXK1	GGAGGAGCAGATCAAGCAGA(F)<br>CAGCACCTCCATCACCTTCT(R)	NM_001137498.1
